# From Bench to Application: Evaluating the In Vitro and In Vivo Efficacy of a Polyhexamethylene Biguanide and Cross-Linked Hyaluronic Acid-Based Antiseptic Solution

**DOI:** 10.3390/jcm14082745

**Published:** 2025-04-16

**Authors:** Francesco D’Oria, Giovanni Petruzzella, Enzo D’Ambrosio, Francesco Pignatelli, Giuseppe Addabbo, Giovanni Alessio

**Affiliations:** 1 Section of Ophthalmology, Department of Translational Biomedicine and Neuroscience, University of Bari, 70121 Bari, Italy; ivan.giovanni.petruzzella@gmail.com (G.P.); giovanni.alessio@uniba.it (G.A.); 2 Centro Oftalmico d’Ambrosio, 74121 Taranto, Italy; dambrosio.enzo@icloud.com; 3 Eye Clinic, “SS. Annunziata” Hospital, ASL Taranto, 74100 Taranto, Italy; pignatelli.oculista@gmail.com (F.P.); giuseppe.addabbo@asl.taranto.it (G.A.)

**Keywords:** polyhexamethylene biguanide (PHMB), cross-linked hyaluronic acid, antiseptic ophthalmic solution, bacterial infections, fungal infections

## Abstract

**Background/Objectives**: In the context of increasing bacterial resistance and the need for effective ophthalmic antiseptics, this study evaluates the antimicrobial efficacy of Corneial MED^®^, a novel ophthalmic solution containing polyhexamethylene biguanide (PHMB) and cross-linked hyaluronic acid. The study investigates the in vitro fungicidal and bactericidal properties of this solution against clinically relevant fungal and bacterial strains and its impact on conjunctival flora in vivo. **Methods**: The in vitro assessment included time-kill assays to determine the fungicidal or fungistatic activity against *Candida albicans*, *Aspergillus flavus*, and *Aspergillus fumigatus*. The bactericidal activity was evaluated against *Staphylococcus aureus* (methicillin-sensitive and -resistant), *Staphylococcus epidermidis*, *Pseudomonas aeruginosa*, and *Escherichia coli*. In vivo, 43 patients undergoing cataract surgery were treated with the solution for three days preoperatively. **Results**: Corneial MED^®^ demonstrated a fungistatic effect against *C. albicans* and *A. fumigatus*, while it exhibited limited activity against *A. flavus*. The tested solution effectively reduced bacterial load within minutes, outperforming competitor ophthalmic solutions in activity against *P. aeruginosa* and *E. coli*. Conjunctival swabs indicated a significant reduction in bacterial load post-treatment, confirming the solution’s efficacy in reducing potential ocular pathogens. **Conclusions**: These findings highlight the potential of PHMB-based antiseptic solutions as a viable alternative to traditional disinfectants, particularly for preoperative prophylaxis and infection control. Further clinical trials are needed to confirm long-term safety and efficacy. The combination with cross-linked hyaluronic acid not only enhances tolerability but also extends antimicrobial action, making it a promising candidate for ophthalmic disinfection.

## 1. Introduction

In an era where ocular infections are becoming an increasingly global threat—one that is mounting due to the appearance of multidrug-resistant bacterial and fungal infections [1]—new and safe therapeutic strategies are urgently needed. The excessive and unjustified use of antibiotics in the treatment of ocular infections has consequently promoted the development of resistance, making it difficult to treat vision-threatening diseases such as conjunctivitis, keratitis, and postoperative endophthalmitis.

Moreover, the scientific debate has highlighted that topical prophylaxis with antibiotics in the perioperative period, despite being widespread, can alter conjunctival flora without offering additional protection compared to antiseptic care, thereby inducing the development of resistant strains [2].

Among antiseptics, polyhexamethylene biguanide hydrochloride (Polyhexanide, PHMB), a cationic biguanide polymer first synthesized by Rose and Swain in 1954 [3], is used in a wide variety of antibacterial applications, due to its excellent antimicrobial activity, chemical stability, low toxicity, and reasonable cost [4]. For this reason, topical preparations with broad-spectrum antiseptics have gained prominence, as they offer effective activity against bacteria [5], fungi [6], viruses [7,8], and protozoa [9,10] while having a lower capacity to induce resistance [11]. In ophthalmic practice, PHMB has been recommended as a preoperative antiseptic treatment for cataract surgery due to its ability to safely and effectively reduce conjunctival flora without causing local or systemic intolerance [12].

The aim of this study is to gather scientific evidence on the bactericidal activity of PHMB both in vitro and in vivo against the conjunctival bacterial flora, which serves as a reservoir for bacteria involved in the onset of endophthalmitis [13]. For this purpose, an ophthalmic solution composed of PHMB 5 ppm and high cross-linked hyaluronic acid was studied in three different settings:

- In vitro to demonstrate its fungicidal or fungistatic activity against three clinically significant fungal isolates commonly responsible for fungal keratitis: *Candida albicans, Aspergillus flavus*, and *Aspergillus fumigatus*.
- In vitro to evaluate its comparative efficacy versus two commercial competitors in terms of bactericidal activity against the following five bacterial strains: *Staphylococcus aureus* ATCC 25923, *Staphylococcus aureus* ATCC 43300, *Staphylococcus epidermidis* ATCC 12228, *Pseudomonas aeruginosa* ATCC 27853, and *Escherichia coli* ATCC 25922.
- In vivo to analyze the ability of CORNEIAL MED^®^ to reduce the bacterial load on the conjunctiva in a clinical setting, a crucial factor in preventing postoperative complications.

The in vitro bactericidal and fungicidal activity experiments were performed in triplicate. Each experiment included both positive and negative controls. So, the number of plates was 92.

CORNEIAL MED^®^ [14] is presented as a new ophthalmic solution that combines the antimicrobial efficacy of polyhexamethylene biguanide (PHMB) 5 ppm with the protective and humectant effects of cross-linked hyaluronic acid, which enhance the contact time with the ocular surface up to six times and has improved physical and chemical stability compared to linear HA [15]. Thus, not only does it facilitate a rapid and specific attack against ocular pathogens, but it also reduces the potential for cytotoxicity conventionally associated with high-dose antiseptics on the ocular surface. This study aims to provide strong scientific evidence supporting the use of this novel ophthalmic solution as a safe and effective method for preventing and treating ocular infections.

## 2. Materials and Methods

The analyzed product, CORNEIAL MED^®^, is an ophthalmic solution designed to provide broad-spectrum antimicrobial protection and adequate corneal hydration with the following composition: PHMB 5 ppm, cross-linked sodium hyaluronate, hydroxypropyl methylcellulose, disodium EDTA, borate buffer, sodium chloride, excipients, and purified water.

### 2.1. Fungicidal or Fungistatic Activity In Vitro

The study utilized three clinical fungal strains (*C. albicans*, *A. flavus*, and *A. fumigatus*), selected based on their sensitivity to itraconazole, as determined by previous antifungal susceptibility testing. All strains were obtained from the ATCC (American Type Culture Collection; Manassas, VA, USA): *C. albicans* (ATCC 10231), *A. flavus* (ATCC 9643), and *A. fumigatus* ATCC 96918).

#### 2.1.1. Experimental Setup

To evaluate the fungicidal or fungistatic activity of CORNEIAL MED, a modified time-kill protocol based on the guidelines from Creative Biolabs was adopted [16,17]. This method tracks the variation in fungal growth over time, expressed as colony-forming units (CFU) per milliliter of culture broth. The ophthalmic solution under study was compared with a positive control (itraconazole) and a negative control (saline solution). Itraconazole was chosen for its fungicidal activity—ascertained via antifungal susceptibility testing—against the three specific clinical isolates tested. This experimental approach allowed the construction of time-kill curves, describing microbial growth trends over time in response to treatment exposure (from 0 to 24 h).

#### 2.1.2. Preparation of Fungal Suspensions and Time-Kill Test

The time-kill method for antifungal activity evaluation was performed by macro-dilution of fungi in a volume of 10 mL, using RPMI 1640 broth buffered with MOPS at pH 7.0 as the culture medium. The fungal suspension was prepared at an initial concentration of 500,000 CFU/mL. Incubation was conducted at 35 °C for 24 h, with constant agitation to ensure optimal growth conditions and homogeneity of the fungal suspension. Serial sampling was performed at predetermined time intervals: at inoculation (time 0) and after 2, 4, 8, 12, and 24 h. Before each sampling event, the suspensions were vortexed to ensure homogeneity. For each time point, a predetermined volume of the suspension (30 µL) was plated on potato dextrose agar (PDA). The plates were incubated at 35 °C for 48 h to allow fungal colonies to grow and to quantify them. The quantification limit of the method was set at 50 CFU/mL. The study included a negative control (saline solution) and a positive control (itraconazole), chosen based on the sensitivity of the isolates as determined in previous antifungal susceptibility tests. The fungal concentrations under different experimental conditions were collected and tabulated at the time of inoculation and at subsequent time intervals (2, 4, 8, 12, and 24 h) for a total of six measurements. Results were interpreted based on the reduction in CFUs. A fungicidal effect was defined as a ≥99% reduction relative to the initial concentration, while a fungistatic effect was attributed to reductions below 99%. This approach allowed a detailed characterization of the antifungal activity—fungicidal or fungistatic—of the tested product in relation to exposure time.

### 2.2. Bactericidal Activity In Vitro

A comparative evaluation was performed on three ophthalmic solutions [CORNEIAL MED, 0.66% povidone-iodine eye drops (IODIM), and ozonized oil eye drops in liposomes (OZODROP)] in terms of in vitro bactericidal activity against bacterial strains commonly isolated in cases of conjunctivitis and endophthalmitis (*S. aureus* ATCC 25923, *S. aureus* ATCC 43300, *S. epidermidis* ATCC 12228, *P. aeruginosa* ATCC 27853, and *E. coli* ATCC 25922). The bactericidal activity of the three ophthalmic solutions was evaluated using the protocol proposed by Berkelman et al. [18], with appropriate modifications and adaptations [19].

#### 2.2.1. Bacterial Strains

The study was conducted using the following bacterial strains, commonly isolated during cases of conjunctivitis and endophthalmitis: *S. aureus* ATCC 25923 (methicillin-sensitive), *S. aureus* ATCC 43300 (methicillin-resistant), *S. epidermidis* ATCC 12228 (methicillin-sensitive), *P. aeruginosa* ATCC 27853, and *E. coli* ATCC 25922. All strains were obtained from the ATCC (American Type Culture Collection; Manassas, VA, USA).

#### 2.2.2. Preparation of Bacterial Suspensions and Bactericidal Activity Test

First, a Standard McFarland 0.5 bacterial suspension was prepared, containing approximately 1.5 × 10^8^ colony-forming units (CFU)/mL for each of the five bacterial strains after overnight growth. A volume of 0.1 mL of the Standard McFarland 0.5 suspension was diluted with 1.9 mL of each of the three ophthalmic solutions under study. Bacterial counts in the suspension/ophthalmic solution mixtures were subsequently evaluated at nine different time intervals (15 s, 30 s; 1, 2, 4, 6, 8 min; 1 and 24 h).

The in vitro bactericidal activity experiments for the three ophthalmic solutions were performed in triplicate, measuring the bacterial load in the mixtures at the following time intervals (compared to the baseline load measured in the absence of ophthalmic solutions): 15 s, 30 s; 1, 2, 4, 6, 8 min; 1 and 24 h. At each of these intervals, the collected mixture was neutralized before bacterial counting by adding 1.9 mL of a 0.5% sodium thiosulfate solution.

Following 24 h incubation at 36 °C, colony counts were manually performed using 0.1 mL of the mixture seeded on TSA (Tryptic Soy Agar) plates (calculated inoculum per species in the absence of ophthalmic solution: approximately 3.75 × 10^3^ CFU/mL). Each experiment included both positive and negative controls.

Bacterial counts for each of the five species, evaluated at the nine different time intervals for each of the three ophthalmic solutions, were expressed as mean and standard deviation.

### 2.3. In Vivo

A total of 43 consecutive patients scheduled to undergo cataract surgery via phacoemulsification were enrolled. Conjunctival swabs were collected from the contralateral eye three days before hospital admission (t0) and immediately prior to surgery (t1). During this period, following the usual protocol, CORNEIAL MED^®^ was instilled three times daily in both eyes. The transport medium was cultured within 24 h according to standard microbiological procedures, and the laboratory provided bacterial colony counts and species identification. A Bayesian Poisson statistical model was developed to analyze variations in bacterial load between t0 and t1, incorporating random effects to account for overdispersion among patients and regularizing priors to compensate for the small sample size.

## 3. Results

### 3.1. Fungicidal or Fungistatic Activity In Vitro

**Figure 1:**
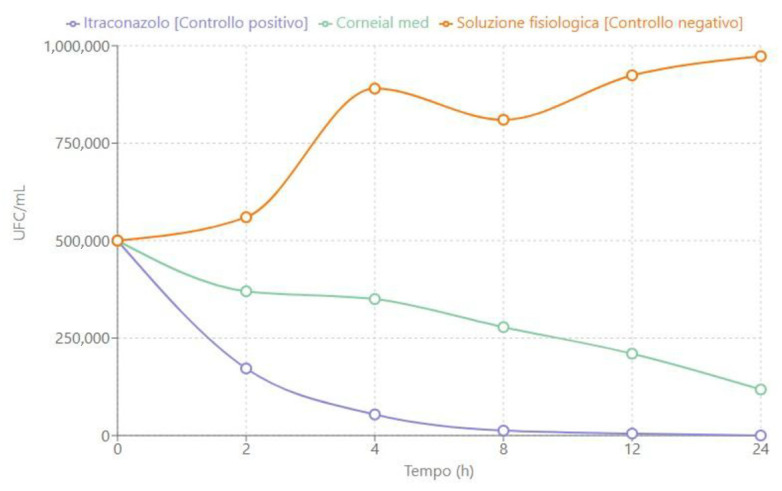
Time-kill curve for a clinical isolate of *C. Albicans* sensitive to itraconazole.

**Figure 2:**
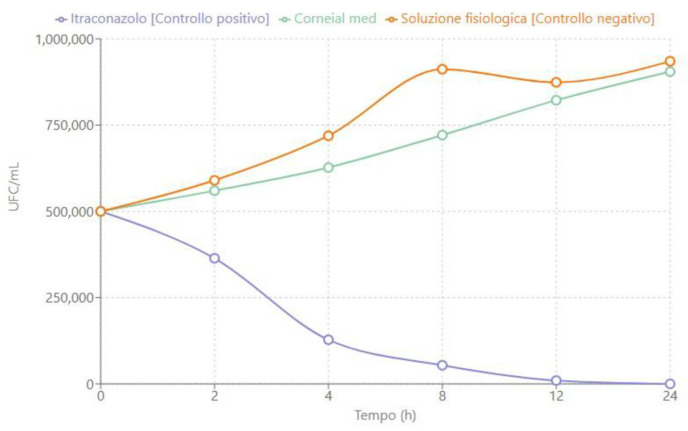
Time-kill curve for a clinical isolate of *A. flavus* sensitive to itraconazole.

**Figure 3:**
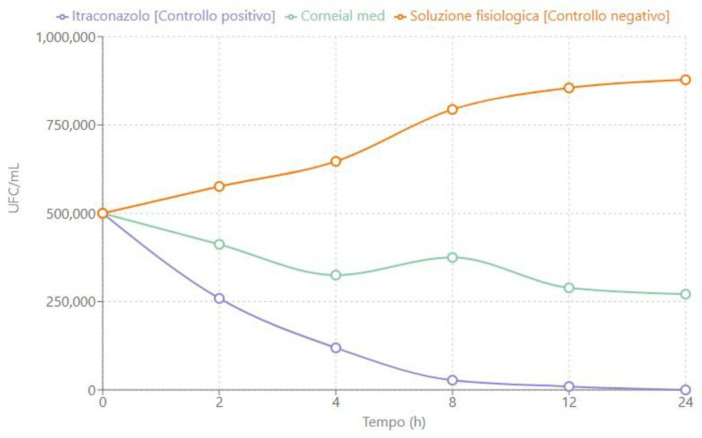
Time-kill curve for a clinical isolate of *A. fumigatus* sensitive to itraconazole.

The time-kill curves shown in Figure 1, Figure 2 and Figure 3 describe the trend of fungal concentration (CFU/mL) over time (hours) for the three treatments tested: itraconazole (positive control), CORNEIAL MED^®^, and saline solution (negative control). As shown, the initial concentration for all groups was 500,000 CFU/mL. The corresponding quantitative data are reported in Table 1, Table 2 and Table 3.

#### 3.1.1. *Candida albicans*

Figure 1 shows the time-kill curve for a clinical isolate of *C. albicans* sensitive to itraconazole. Itraconazole—as expected—demonstrated rapid fungicidal activity, with a progressive reduction in fungal load, reaching a value below the limit of quantification (<50 CFU/mL) within 24 h.

CORNEIAL MED^®^ showed a fungistatic effect, with a significant reduction in fungal concentration, decreasing from 500,000 CFU/mL to approximately 118,000 CFU/mL after 24 h. In contrast, the saline solution exhibited an increase in fungal load, reaching a final value of 973,000 CFU/mL within the same time frame (Table 1).

**Table 1:** Fungal concentration (CFU/mL) of *C. albicans* over time.

Time (h)	Itraconazole [Positive Control]	Corneial Med	Negative Control
0	500,000	500,000	500,000
2	172,000	370,000	560,000
4	54,000	350,000	890,000
8	12,500	278,000	810,000
12	5000	210,000	924,000
24	<50	118,000	973,000

#### 3.1.2. *Aspergillus flavus*

Regarding *A. flavus*, Figure 2 shows that itraconazole once again demonstrated fungicidal efficacy, reducing the initial concentration from 500,000 CFU/mL to less than <50 CFU/mL within 24 h.

CORNEIAL MED^®^ showed a limited effect, with a gradual increase in fungal load, reaching a final value of approximately 905,000 CFU/mL at the end of the 24 h observation period. Similarly, the saline solution exhibited steady and marked growth, with a final concentration of approximately 935,000 CFU/mL (Table 2).

**Table 2:** Fungal concentration (CFU/mL) of *A. flavus* over time.

Time (h)	Itraconazole [Positive Control]	Corneial Med	Negative Control
0	500,000	500,000	500,000
2	364,000	560,000	590,000
4	128,000	627,000	719,000
8	54,000	721,000	912,000
12	9800	822,000	874,000
24	<50	905,000	935,000

#### 3.1.3. *Aspergillus fumigatus*

In the case of *A. fumigatus*, as shown in Figure 3, itraconazole confirmed its fungicidal activity, reducing the initial concentration from approximately 500,000 CFU/mL to less than <50 CFU/mL within the first 24 h of incubation. (Table 3).

**Table 3:** Fungal concentration (CFU/mL) of *A. fumigatus* over time.

Time (h)	Itraconazole [Positive Control]	Corneial Med	Negative Control
0	500,000	500,000	500,000
2	259,000	412,000	576,000
4	119,000	325,000	647,000
8	27,500	375,000	794,000
12	9700	289,000	855,000
24	<50	271,000	878,000

### 3.2. Bactericidal Activity In Vitro

**Figure 4:**
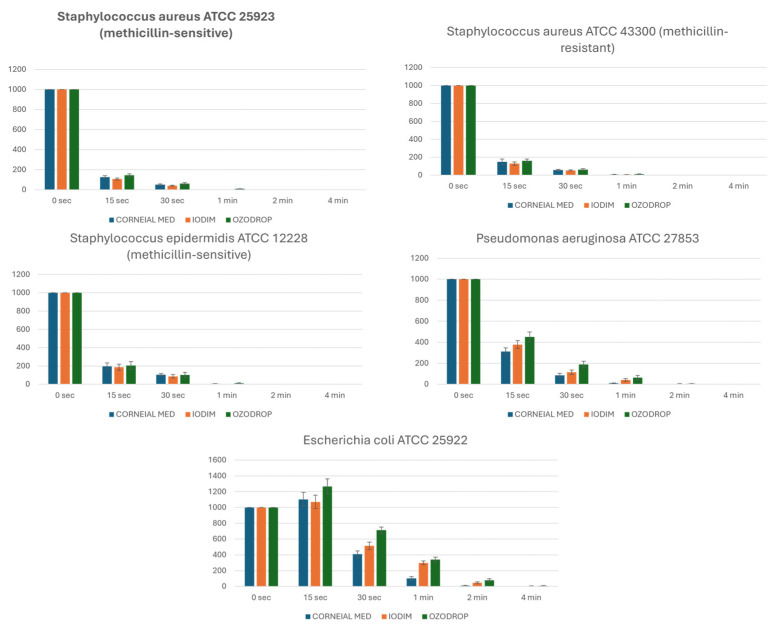
Comparative time-kill curves of three ophthalmic solutions (CORNEIAL MED, IODIM, OZODROP) against standard strains of *S. aureus* (methicillin-sensitive and -resistant), *S. epidermidis*, *P. aeruginosa*, and *E. coli*.

The results of bacterial counts obtained at different time intervals from 0.1 mL of standard bacterial suspension mix with the three ophthalmic solutions are reported in Table 4 and Figure 4. Complete bactericidal activity (bacterial count: 0) was observed for all three ophthalmic solutions at 6 and 8 min, as well as at 1 and 24 h. Therefore, these data were not tabulated.

### 3.3. In Vivo

Of the 43 patients initially enrolled, 2 were lost before t0. At t0, 34% of the samples tested positive for bacterial presence. Following treatment, all patients except one tested negative. Five patients who were initially negative at t0 became positive at t1, and one patient, although negative for the original bacterial strain, tested positive for *S. aureus* at t1. The Bayesian statistical model indicated a 99.9% probability that, in cases of initial bacterial contamination, there was a reduction in bacterial load following treatment. The mean bacterial load reduction was estimated at 97% [CrI: 82–99%], with a greater reduction observed in cases with higher initial bacterial loads at t0.

## 4. Discussion

This study demonstrated that CORNEIAL MED^®^, an ophthalmic solution containing PHMB, is capable of exerting effective fungistatic activity against *C. albicans* and *A. fumigatus*, although it did not show significant inhibitory effects against *A. flavus*. Conversely, itraconazole—used as a positive control based on the sensitivity of the three strains to this drug (confirmed through prior antifungal susceptibility testing)—consistently exhibited marked fungicidal activity against all three fungal species tested, reducing their burden to unquantifiable levels within 24 h. Saline solution, on the other hand, allowed a highly significant growth of fungal burden for all three species examined, without exhibiting any biological activity, consistent with its use as a negative control.

The fungistatic activity of CORNEIAL MED^®^ against *C. albicans* and *A. fumigatus* confirms the potential of PHMB—a cationic polymer with a broad spectrum of antimicrobial activity—as an antifungal agent [11,20]. It is believed that this compound primarily acts by altering the integrity of the fungal cell membrane, causing a loss of functionality and destabilization of intracellular structures [21]. Its efficacy against *C. albicans* is particularly noteworthy, as this species is a common etiological agent of fungal keratitis, and the ability to limit its proliferation may represent a valid therapeutic option in specific clinical contexts. The fungistatic effect observed against *A. fumigatus* also appears promising, consistent with the action observed against *C. albicans*.

Antibiotic resistance, which can complicate the choice of antibiotic in clinical practice and in some cases lead to treatment failure, is a growing concern that can make it difficult to effectively treat patients with eye infections [22]. In fact, although antibiotic therapy has improved the rates of clinical remission in patients suffering from eye infection [23], microbial resistance to antibiotics continues to be a serious problem that is on the rise and needs to be overcome [24]. Thus, an attempt should be made to identify the pathogen responsible for the ocular infection, performing the antibiotic susceptibility testing and analysis as soon as possible and avoiding the indiscriminate use of broad-spectrum antibiotics in preoperative treatment, favoring antiseptic solutions. In the ARMOR 2013 study, 86.8% and 77.3% of MRSA and methicillin-resistant CoNS, respectively, were found to be resistant to three or more drug classes [25].

Regarding the bactericidal and bacteriostatic effects, when compared with two commercially available ophthalmic solutions, all of them demonstrated substantially similar activity against Staphylococcus aureus ATCC 25923 (methicillin-sensitive) and *S. aureus* ATCC 43300 (methicillin-resistant), with bacterial count negativization at 2 and 4 min for all tested products. A similar result was observed for *S. epidermidis* ATCC 12228 (methicillin-sensitive), for which the first product to achieve negativization at 1 min was Iodim. Corneial Med outperformed its competitors regarding activity against *P. aeruginosa* ATCC 27853 and *E. coli* ATCC 25922. Specifically, for *P. aeruginosa* ATCC 27853, Corneial Med achieved negativization at 2 min, while Iodim and Ozodrop achieved it only at 4 min. For *E. coli* ATCC 25922, Corneial Med achieved negativization in just 4 min, whereas Iodim and Ozodrop achieved it after 6 min. The faster action of Corneial Med against the highly pathogenic bacterium Pseudomonas aeruginosa is likely attributable to the ability of the cationic polymer PHMB to inhibit elastase produced by this microorganism [26], which represents a significant ocular virulence factor [27]; the faster action of Corneial Med against *E. coli* is probably due to PHMB’s ability to bind and rapidly precipitate the bacterium’s DNA [28].

In conclusion, the disinfectant activity of PHMB has been well documented, and the need to reduce antibiotic use is urgent. This pilot study provides evidence that PHMB, even at a concentration of 5 ppm, retains its disinfectant capacity in vivo and significantly reduces the conjunctival bacterial load, a potential source of infectious complications. Moreover, the combination with crosslinked hyaluronic acid not only ensures excellent tolerability but also prolongs the retention of the active ingredient on the ocular surface, thereby enhancing its efficacy. These findings lay the groundwork for further in vivo studies to confirm the utility of CORNEIAL MED^®^ as a disinfectant, not only for the treatment of infectious diseases but also for prophylactic use in refractive surgery, cataract surgery, intravitreal injections, and other ophthalmic procedures. Further studies are needed to evaluate its effectiveness over extended periods and to assess its relative effectiveness in a broader clinical context with other existing antiseptic solutions or antibiotics.

## Figures and Tables

**Table 4:** Comparison of bactericidal activity of ophthalmic solutions against various bacterial strains (time-kill assay results).

Strain	Product	0 s	15 s	30 s	1 min	2 min	4 min
*Staphylococcus aureus* ATCC 25923 (methicillin-sensitive)	CORNEIAL MED	>103	127 ± 12	51 ± 7	0	0	0
	IODIM	>103	107 ± 10	41 ± 5	0	0	0
	OZODROP	>103	144 ± 15	62 ± 10	9 ± 2	0	0
*Staphylococcus aureus* ATCC 43300 (methicillin-resistant)	CORNEIAL MED	>103	148 ± 32	58 ± 8	7 ± 3	0	0
	IODIM	>103	129 ± 19	52 ± 6	5 ± 2	0	0
	OZODROP	>103	159 ± 20	62 ± 11	10 ± 4	0	0
*Staphylococcus epidermidis* ATCC 12228 (methicillin-sensitive)	CORNEIAL MED	>103	195 ± 38	105 ± 11	5 ± 2	0	0
	IODIM	>103	186 ± 34	87 ± 19	0	0	0
	OZODROP	>103	204 ± 42	101 ± 29	11 ± 5	0	0
*Pseudomonas aeruginosa* ATCC 27853	CORNEIAL MED	>103	311 ± 35	84 ± 19	10 ± 2	0	0
	IODIM	>103	378 ± 38	115 ± 21	41 ± 13	4 ± 2	0
	OZODROP	>103	451 ± 47	188 ± 32	63 ± 20	5 ± 2	0
*Escherichia coli* ATCC 25922	CORNEIAL MED	>103	1103 ± 87	408 ± 42	102 ± 24	11 ± 4	0
	IODIM	>103	1071 ± 84	515 ± 47	299 ± 24	47 ± 11	5 ± 3
	OZODROP	>103	1267 ± 95	712 ± 39	341 ± 29	78 ± 20	8 ± 4

## Data Availability

Data are available on request.

## Abbreviations

The following abbreviations are used in this manuscript:

PHMB	Polyhexamethylene biguanide
CFU	Colony-forming unit
